# The Control of Measles: The Value, as a Safeguard, of a Mild Epidemic

**Published:** 1913-07-12

**Authors:** 


### THE CONTROL OF MEASLES.

### The Value, as a Safeguard, of a Mild Epidemic.

FROM A MEDICAL CORRESPONDENT.

An interesting discussion on the control of measles was recently held by the Medical Officers of Schools Association, and the views thereat enunciated deserve to be brought prominently before parents and the public generally. Whenever the lay press gives notice of the fact that an outbreak of the disease has occurred at some public school there appear letters, emanating mostly from laymen who are imperfectly acquainted with the course and history of the disease, and who frankly, and perhaps too faithfully, accept the dictum that measles is one of the most fatal of all diseases that afflict childhood, calling for more vigorous control of the disease. There is undoubtedly among laymen a desire that all epidemics should be stopped. Some, indeed, regard as an ideal a community in which measles is unknown, as smallpox is comparatively unknown. But these enthusiasts entirely fail to regard the facts squarely. They postulate that since typhus has been vanquished and smallpox almost eradicated, it is only a matter of work and time before measles and scarlet fever are similarly extinguished. Against this, the obvious argument is that the comparison is not a fair one. Smallpox, for instance, has been rendered innocuous largely through the employment of prophylactic vaccination; typhus through improved sanitation. But in the case of measles and scarlet fever we know that one attack confers immunity; not absolute, it is true, but sufficient, indeed, to be accepted as a safeguard to the rest of the community. A race entirely free from epidemics is likely to be in the same unfavourable condition in which the South Sea islanders were found when the first measles epidemic overtook them. Until we have found a satisfactory means of inoculating or vaccinating against the disease, we must, therefore, look upon a mild epidemic of measles as a safeguard, and not wholly as a danger to the community, since it confers on those affected that immunity which they need. The whole matter of the control of the disease, therefore, resolves itself into a question of controlling the epidemics as they arise, and keeping them within due limits. Most of the speakers at the discussion above alluded to favoured this view; some, like Dr. Lempriere, of Haileybury College, asserted that control of measles is non-existent, and that given one case an epidemic will inevitably follow, while, even if control is possible, it would not be in the best interests of the community to enforce it. Those who have had most experience believe, with much justification for that supposition, that measles in a community of children between nine and thirteen is much less serious than in a community of older children.

The influence of the school is still said to be one of the great factors favouring the dissemination of measles. This inference is against the mass of observation of recent date. Indeed, the conclusions to be drawn from the study of statistical evidence available seem to be that school closure has but little effect in preventing an epidemic. Dr. Kerr gave a case of a girl of five who was known to have been the cause of distributing the disease to no fewer than 207 children, necessitating fourteen class closures in four different schools. Home conditions and out-of-school environment undoubtedly are far more potent factors in spreading the disease than are the schools, and though school closure is still insisted upon by some medical officers of health, it is highly doubtful if such closure is called for in the interests of the community.

A point to be referred to in any discussion of the subject is the treatment of cases. Measles is an ailment which is attended by grave complications and sequelæ. Prompt and careful treatment, therefore, is necessary in the slightest case. It is argued that to make the disease notifiable in all districts is to entail extra expense, which the results do not justify. This is overstating the case against notification, for it is problematical whether medical officers of health have hitherto made the best use of notifications. Equally divergent are the views with regard to disinfection. It is admitted that whatever is the virus that causes the disease, it is of low vitality; though highly infectious in the first stages, it is rapidly destroyed and not carried about by fomites. But, as Dr. Dudfield pointed out, "even if disinfection be rated at a minimum of utility, there still remain two advantages to be gained by its use." In the first place, it secures in the poorer homes a measure of subsequent cleanliness, and, secondly, it impresses "on the minds of the public the view that the sanitary authority holds the disease as one which is not to be regarded with a careless indifference, either as regards the patient or the public generally." This treatment of the subject strikes us as being the common-sense one. Without the help of the public and the parent, the medical officer is seriously handicapped in trying to cope with the disease. The parent should be educated up to the importance of notifying the teacher; the teacher, in turn, to the desirability of notifying the authority. With such help, derived from immediate notification, the authorities are in a position to deal with cases as they occur, without waiting for an epidemic to occur. As Dr. Shelly, in summing up the discussion remarked, the policy in the future must be chiefly to protect the very young from infection until the milder, unmixed, infection breeds true, and then to "encourage repeated small epidemics at the age of about seven to eight years, until the public is educated to accept inoculation or vaccination." This seems, to the lay mind at least, an amazing policy to pursue, but it is likely, from the evidence, to be the only one from which the community can expect general benefit.

